# Broad immunogenicity of house dust mite proteins contrasts restricted specific IgE and IgG4 associated with allergy

**DOI:** 10.1371/journal.pone.0338593

**Published:** 2026-02-09

**Authors:** Lars Harder Christensen, Jens Emil Vang Petersen, Gitte Lund, Jens Holm, Peter Adler Würtzen, Jakob Wiborg, Henrik Ipsen, Niels Johansen, Thomas Stranzl, Peter Sejer Andersen

**Affiliations:** Global Research & Drug Discovery, ALK-Abelló A./S., Hørsholm, Denmark; Universidade Federal do Rio de Janeiro, BRAZIL

## Abstract

**Background:**

House dust mite (HDM) allergy involves IgE and T_H_2 responses to major and minor allergens. Less is known about the involvement of other immune pathways and the potential role of other HDM proteins in allergic disease. In this study, the association between HDM allergy and immune responses to the HDM proteome was investigated.

**Methods:**

The HDM proteome was represented by 40 purified recombinant HDM proteins (19 known allergens and 21 novel proteins). T-cell responses to HDM proteins were determined *ex vivo* and antibody responses (IgA, IgE, IgG and IgG4) were measured using micro arrays and basophil activation in 21 HDM allergic donors and 16 non-allergic controls. Changes in specific IgE, IgG and IgG4 during SQ HDM SLIT-Tablet immunotherapy was assessed in 38 subjects with allergic asthma.

**Results:**

HDM proteins were broadly immunogenic inducing comparable IgG, IgA, and non-T_H_2 cytokine responses in both allergic and non-allergic individuals. Specific IgE, IgG4 and T_H_2 cytokine responses were largely restricted to the allergic donors. IgE and IgG4 were primarily directed to known major allergens and overlapping in specificity whereas cellular T_H_2 responses extended beyond the known HDM allergens. Individual proteins displayed distinct immunological profiles. HDM sublingual immunotherapy increased the levels of specific IgE and IgG4 but did not change the overall pattern of recognition.

**Conclusion:**

HDM proteins are highly immunogenic and give rise to complex patterns of immune recognition also in the absence of allergy. This has potential implications for the pathogenesis of HDM allergy and the mode of action of allergy immunotherapy.

## Introduction

House dust mite (HDM) allergy is the most common allergy worldwide affecting an estimated 65–130 million people, corresponding to a prevalence of 1–2% of the global population [1]. The two most common HDM species *Dermatophagoides pteronyssinus* (Der p) and *Dermatophagoides farinae* (Der f) are responsible for more than 90% of HDM allergies worldwide [2]. Their allergens are homologous (80–85% sequence identity) with extensive IgE and T-cell cross-reactivity [3,4].

In addition to HDM-derived T-cell epitopes of the major allergens such as Der p/f 1 and Der p/f 2 [4–7], T-cell epitopes capable of inducing both Th2 and Th1 polarized T-cell responses, have been identified in HDM proteins beyond the official allergens [8].

Of the current 44 official IUIS/WHO (www.allergen.org) groups of HDM allergens, Group 1, 2, 23, and 35 are classified as major allergens with specific IgE prevalence above 50% in HDM allergic individuals [9–11]. Group 4, 5, 7, 13, 15 and 21 are frequently reported minor allergens, prevalent in 20–40% [12]. Sensitization to the remaining minor allergens is less prevalent. Typically, more than 50% of the total HDM-specific IgE is towards group 1 and 2 [12,13]. Unlike HDM specific IgE levels that are significantly higher in HDM allergic individuals compared to non-allergic, HDM-specific IgA levels have been found to be higher in non-allergic individuals compared to allergic individuals [14]. How variations in IgE recognition of allergens relates to HDM-specific antibody repertoires of other isotypes has not been investigated in detail.

Allergy immunotherapy (AIT) such as tablet based sublingual immunotherapy (SLIT) or subcutaneous immunotherpay (SCIT) is a disease-modifying treatment that confer long-term clinical benefit to pollen [15,16] and HDM [17] allergy following discontinuation of treatment, and effectively reduces asthma exacerbations and medicine use in HDM allergic asthma [18–20]. SLIT consistently increases IgE-blocking antibodies, particularly the IgG4 isotype, and blocking of IgE binding likely contributes to the tolerogenic mechanism behind the sustained effect [21]. Knowledge about how HDM SLIT affects IgE sensitization patterns to individual HDM components is limited to major allergens [13].

To address the immunogenicity of HDM proteins more widely, we profiled HDM allergic individuals and individuals without HDM allergy by comparing antibody and T-cell responses to a comprehensive panel of individual recombinant HDM proteins (19 allergens and 21 novel proteins). Further, changes in antigen recognition patterns of IgG4 and IgE during SQ HDM SLIT-Tablet treatment was assessed post-hoc in a subgroup of patients from a clinical trial with HDM allergic asthma.

## Materials and methods

### Identification of novel HDM proteins by mass spectrometry

Novel HDM proteins were identified from 10 min aqueous extractions of Der p and f body and fecal particles by LC-MS/MS searched against in-house HDM transcriptomes as previously described [8].

### Expression/purification of recombinant HDM proteins

A total of 19 known (IUIS) HDM allergens and 21 novel, uncharacterized HDM proteins, derived from in-house transcriptomics/proteomics [8] were expressed recombinantly in-house in HEK293 or *P. pastoris,* or obtained from Genscript (Rijswijk, Netherlands) expressed in *E. coli* or Sf9 (Table S1 in S1 File). Proteins were His tagged and purified via immobilized metal affinity chromatography (SDS page in Figure S1 in S1 File). The recombinant proteins were formulated in PBS pH 7.4 and free of endotoxins (≤0.01 EU/μg protein). All single HDM proteins included in this study have been confirmed to be present within the SQ HDM SLIT tablet drug substance by LC-MS (data not shown).

### Study population

In Denmark, blood samples were collected from 21 individuals aged ≥18 years with a clinical diagnosis of HDM allergy and Der p and Der f extract IgE titers of ≥0.35 kUA/L. Blood samples were also taken from 16 control individuals aged ≥18 years, diagnosed as not having HDM allergy after consultation with a physician with no symptoms of allergy or allergic rhinitis being reported and thus considered non-allergic. These non-allergic individuals were not excluded based on specific IgE levels towards HDM extracts.

From the MITRA trial—a randomized, double-blind, placebo-controlled study of subjects aged ≥18 years with HDM-induced asthma and allergic rhinitis, with or without conjunctivitis across 13 countries [19] -samples from 38 individuals receiving 12 SQ HDM SLIT-tablet treatment with detectable IgG4 levels, and 20 individuals receiving placebo with detectable specific IgG4 (Der p/f specific IgG4 above limit of quantification of 0.07mgA/L) were retrospectively analyzed for serum antibody levels against the panel of HDM proteins both at baseline and after 9–12 months of treatment.

### Ethics statement

The sample collection was reviewed and approved by the Danish ethics committee (protocol number H-16038444 & H-3-2014-129). The MITRA trial was reviewed and approved by the relevant ethics committees and institutional review boards (Trial number NCT01433523, clinicaltrialsregister.eu identifier: 2010-018621-19). Written consent was obtained from all study participants. Participant information was handled according to GDPR regulations, and final data was extracted from samples the 14^th^ of April 2021.

### Antibody profiling

Recombinant HDM proteins and antibody iso-type controls, and negative controls (BSA and rHSA) were printed in triplicates on custom made protein arrays (Raybiotech, GA, USA). Each serum sample was tested on four identical arrays with different detection against IgA, IgE, IgG and IgG4. Fluorescent signal intensities were measured after excitation at 532 nm (RayBiotech). Lower limit of quantification was defined as the mean antibody signal to BSA plus 10 standard deviations. Specific IgE and IgG4 levels towards Der p extract were quantified using ImmunoCAP (Thermo Fisher, MA, USA).

### Ex vivo basophil activation test and cytokine induction

Basophil activation and cytokine measurements were performed as previously described [22,23]. Briefly, blood samples were incubated with HDM proteins in RPMI supplemented with serum and fluorochrome conjugated anti-CD63 (Immunotech, cat. number IM1165), anti-CD203c (Biolegend, cat. number 324610), and anti-CD123 (BD Biosciences, cat. number BD340545). Activation, i.e., increase in CD63 positive cells), was measured using BD LSRFortessa™ flow cytometer (BD Biosciences) and FlowJo™ (ver.10.7.1 BD Bioscience) was used for analysis.

For *ex vivo* cytokine induction, PBMCs were isolated using Leucoseptubes (Greiner, Art. No: 227290) with 15mL Lymfoprep (Fresenius Kabi, cat. number 1114547). In brief, up to 30 ml of blood was centrifuged for 15 minutes at 765 g with minimal brake, and the cell layer was washed with 2x volume PBS. The cells were then centrifuged for 20 minutes at 300 g, and the pellets were washed twice for 10 minutes at 300 g. Cell pellets were resuspended and adjusted to 5 x 10^5 cells/well in 100 µl volume of RPMI (RPMI 1640 Gibco 72400−021) supplemented with 5% AB serum (Sigma H4522), Pen/strep (Lonza DE17-602E), and then incubated with HDM proteins for 5 days at 37°C, 5% CO_2_. A Meso Scale assay using the U-plex kit U-plex Biomarker group 1: Cat K15067L-4 was used to measure IFNγ, IL-10, IL-13, IL-17A, IL-5, IL-9, and TNF-α according to manufacturer’s protocol. As determined by titration, majority of HDM proteins were used at 10 µg/ml. Der p 2, Der p 1 and Der p 23 were used at 2 µg/ml. N10, N16 and N19, were used at 2 µg/ml and 0.4 µg/ml. Maximal cytokine release is reported for each protein.

### Statistical analysis and visualization

All statistical analyses and visualizations were performed using R [24] with packages “Tidyverse” [25] and ”Arsenal”. Pearson’s Chi-square test was used to compare frequencies, and Kruskal Wallis rank sum test and Dunn’s test was used to compare levels. Alpha values below 0.05 were considered statistically significant, and Bonferroni correction was used to adjust for multiple comparisons. For correlations, Kendall rank correlation coefficient, tau-b, was calculated to account for ties and non-normally distributed data, correlation with tau-b > 0.06 was considered weak, > 0.26 moderate, > 0.49 strong, > 0.71 very strong.

Unsupervised hierarchical clustering on the individual allergens was performed on Z-score normalized values across assays using the “pheatmap” R package.

## Results

### House dust mite proteins are inherently broadly immunogenic in allergic and non-allergic individuals

Based on our previous study characterizing the T-cell epitopes of known and novel HDM proteins identified by mass spectrometry and using synthetic peptides [8], we expressed 19 known allergens and 21 novel proteins (Table S1 & S2 in S1 File), and investigated levels of specific antibodies and ability to induce T-cell responses in a cohort of 21 physician-diagnosed HDM allergic donors and 16 non-allergic control donors (Table S3 in S1 File.

**Fig 1:**
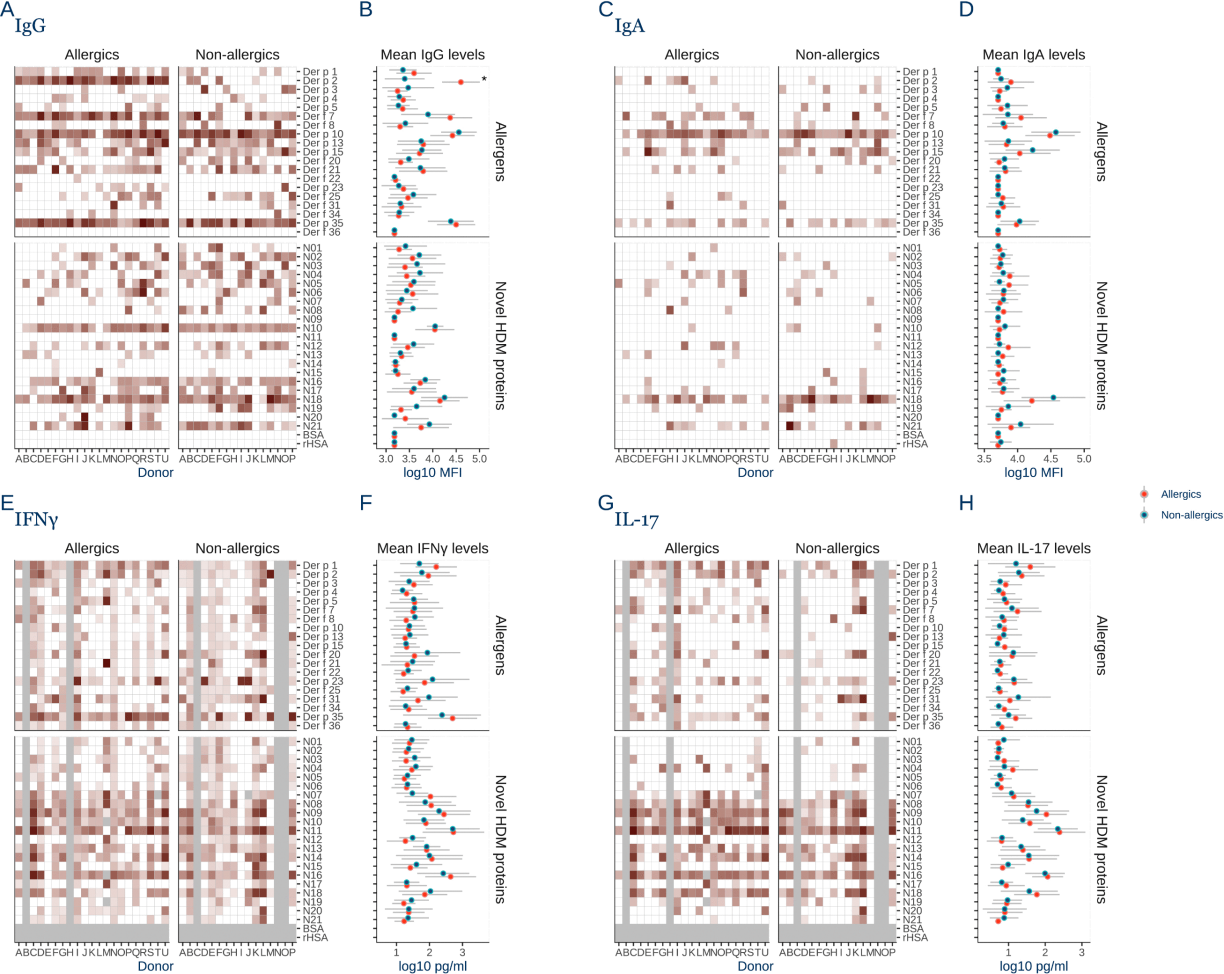
House dust mite proteins are highly immunogenic independent of allergy. **A)** Heatmap of IgG levels against 40 house dust mite (HDM) proteins plus negative controls (BSA+ rHSA) in allergic donors (n = 21) and non-allergic donors (n = 16). Log 10 transformed fluorescence intensity measured in triplicates in a micro array immunofluorescent assay. **B)** Log 10 transformed mean fluorescent intensity (MFI) IgG levels for the individual HDM proteins are shown for allergic donors in red and for non-allergic donors in blue. C) Heatmap of IgA levels against the HDM proteins in allergic and non-allergic donors. **D)** Mean IgA levels towards the individual proteins. **E)** Heatmap of IFNγ induction by the HDM proteins in allergic and non-allergic donors. IFNγ from donor PBMCs stimulated for 5 days with individual HDM proteins quantified in a Uplex assay. **F)** Mean IFNγ response to each HDM protein for allergic and non-allergic donors. **G)** Heatmap of IL-17 induction by the HDM proteins in allergic and non-allergic donors measured with in a Uplex assay. **H)** Mean IL-17 response to each HDM protein for allergic and non-allergic donors. Error bars show the standard deviation of the log transformed values. Statistical significance was evaluated with Kruskal Wallis test, and Dunn’s post hoc test with Bonferroni correction and p-values >0.05 are indicated by *. Grey tiles denote missing data.

Overall, both allergic and non-allergic donors exhibited broad IgG recognition of HDM proteins, with significant variation between individual HDM proteins (Fig 1A). Only specific IgG against Der p 2 was found in more allergic compared to non-allergic donors (100% vs 25% respectively, P < 0.001), and at higher mean levels (P < 0.001) (Fig 1B). Der p 1 was not significantly different (P = 0.13). Der f 7, Der p 10, Der p 35, N10, and N18 were all broadly recognized by IgG in 75–100% of donors, independent of allergy status.

Compared to IgG, fewer HDM proteins were recognized by IgA, with no differences between allergic and non-allergic donors (Fig 1C-D). Der p 10 was the most recognized protein by specific IgA (90% allergic and 100% non-allergic donors), followed by N18 (67% allergic, 94% non-allergic).

T-cell cytokines IFNγ and IL-17 induction was similar between both donor groups (Fig 1E-H). N10 was the top inducer of IFNγ and IL-17, while Der p 35 induced a strong IFNγ response. On average, novel HDM proteins induced more IFNγ and IL-17 than known allergens (P < 0.001).

In summary, both allergens and novel HDM proteins are broadly non- T_H_2 immunogenic, irrespective of allergy disease status.

### Overlapping specificity of IgE and IgG4 responses

**Fig 2:**
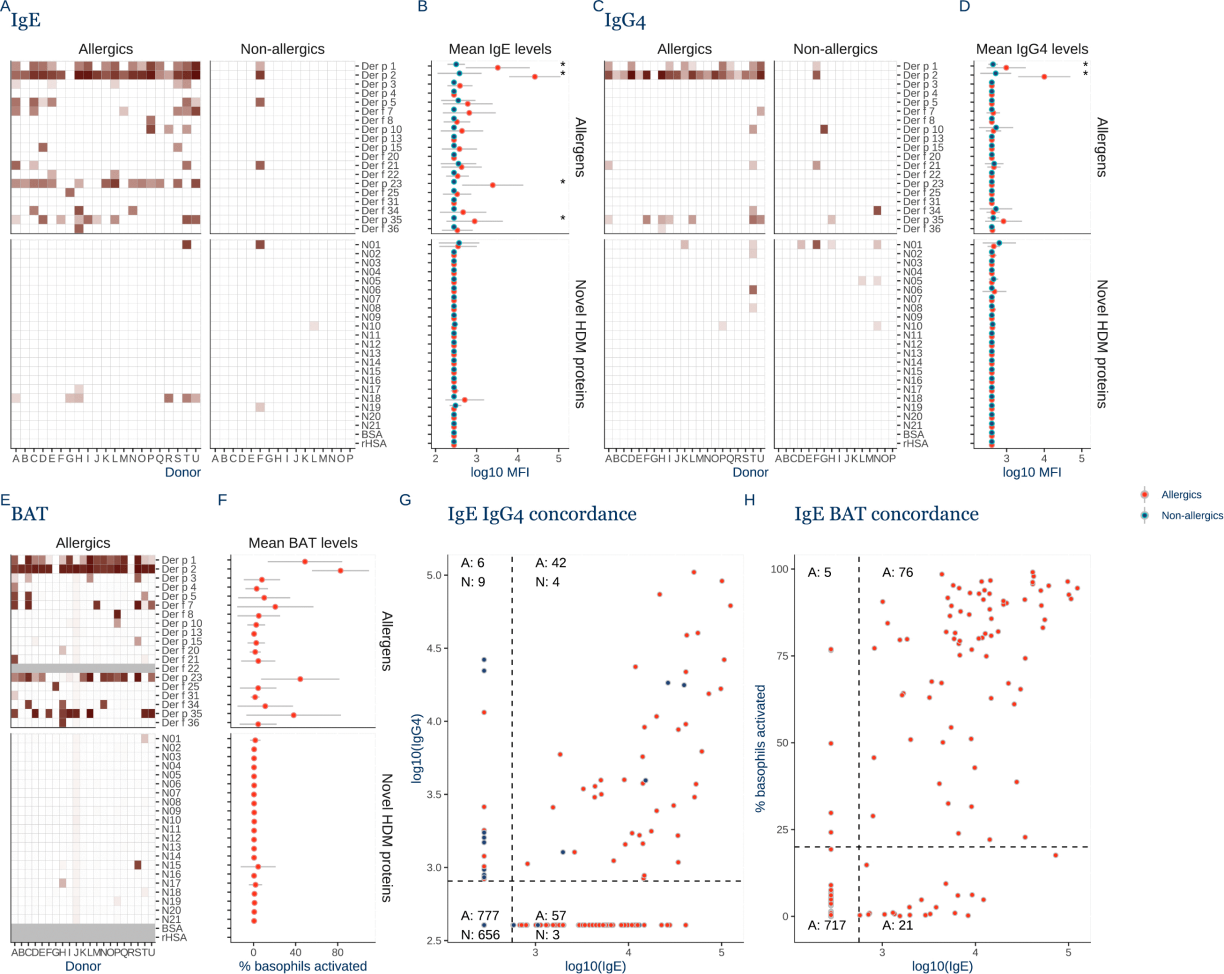
Overlapping and restricted specificity of IgE and IG4 in HDM allergy. **A)** Heatmap of IgE levels against 40 house dust mite (HDM) proteins in allergic donors (n = 21) and non-allergic donors (n = 16, n = 14 for the BAT assay) measured in triplicates in an immunofluorescent micro array-based assay. **B)** Log 10 transformed mean fluorescent intensity (MFI) IgE levels for the individual HDM proteins are shown for allergic donors (red) and for non-allergic donors (blue). **C)** Heatmap of IgG4 levels against the HDM proteins in allergic and non-allergic donors. **D)** Mean IgG4 levels towards the individual proteins. Error bars denote the standard deviation of the log transformed values. Statistical significance was evaluated with Kruskal Wallis test, and Dunn’s post hoc test with Bonferroni correction and p-values >0.05 are indicated by *. **E)** Heatmap of Basophil activation test (BAT) measured as percent of basophil cells with positive surface levels of CD63 in flow cytometry after stimulation with HDM proteins. Missing data is grey. **F)** Mean Basophil activation for each HDM protein. **G)** Concordance between specific IgE and IgG4 to HDM proteins for allergic donors (red) and for non-allergic donors (blue). **H)** Concordance between IgE and basophil activation. Individual log normalized IgE measurements and BAT percentages are shown for allergic donors as red dots. The lower limit of quantification marked by dashed lines is defined as the BSA value plus 10 standard deviations.

In contrast, we found restricted specific IgE towards Der p 1 in 71%, Der p 2 in 95%, Der p 23 in 67% and towards Der p 35 in 43% of allergic donors (Fig 2A), with significantly higher mean IgE levels compared to non-allergic donors (P < 0.01) (Fig 2B). Specific IgE to minor allergens was less prevalent. Furthermore, sporadic IgE to the novel proteins, notably N18 (6/21), indicates that IgE sensitization extends beyond the current official IUIS list of 44 allergens. A single (1/16) non-allergic (asymptomatic) donor (donor F) had IgE towards Der p 1 and 2.

We predominantly observed specific IgG4 in allergic donors, where 42% had IgG4 to Der p 1, 95% to Der p 2, and 33% to Der p 35 (Fig 2C). However, only Der p 1 and Der p 2 had statistically significantly higher levels than non-allergic donors (P < 0.01) (Fig 2D). IgG4 to Der p 23 was absent. IgG4 to Der p 1 and 2 was also present in the IgE positive asymptomatic non-allergic donor (donor F).

We assessed IgE functionality in the allergic donors through basophil activation and observed activation (20% or more CD63-positive basophils) for Der p 1 in 71%, to Der p 2 in 90%, to Der p 23 in 62%, and to Der p 35 in 43% of the allergic donors, mirroring the restricted IgE response (Fig 2E &F).

IgG4 largely overlapped with IgE: Of the 48 specific IgG4 positive instances in allergic individuals, 42 (90%) were also positive for specific IgE (Fig 2G). Similarly, 76/81 (93%) of the instances of basophil activation above 20%, were accompanied by positive specific IgE (Fig 2H), which indicates functional and polyclonal IgE antibody responses to all individual IgE-positive allergens and novel proteins.

Together, the natural sensitization of subjects with overlap between specific IgG4 and IgE points to IgG4 synthesis as an integral component of the allergic sensitization.

### Broad T_H_2 response to HDM proteins is restricted to allergic individuals

**Fig 3:**
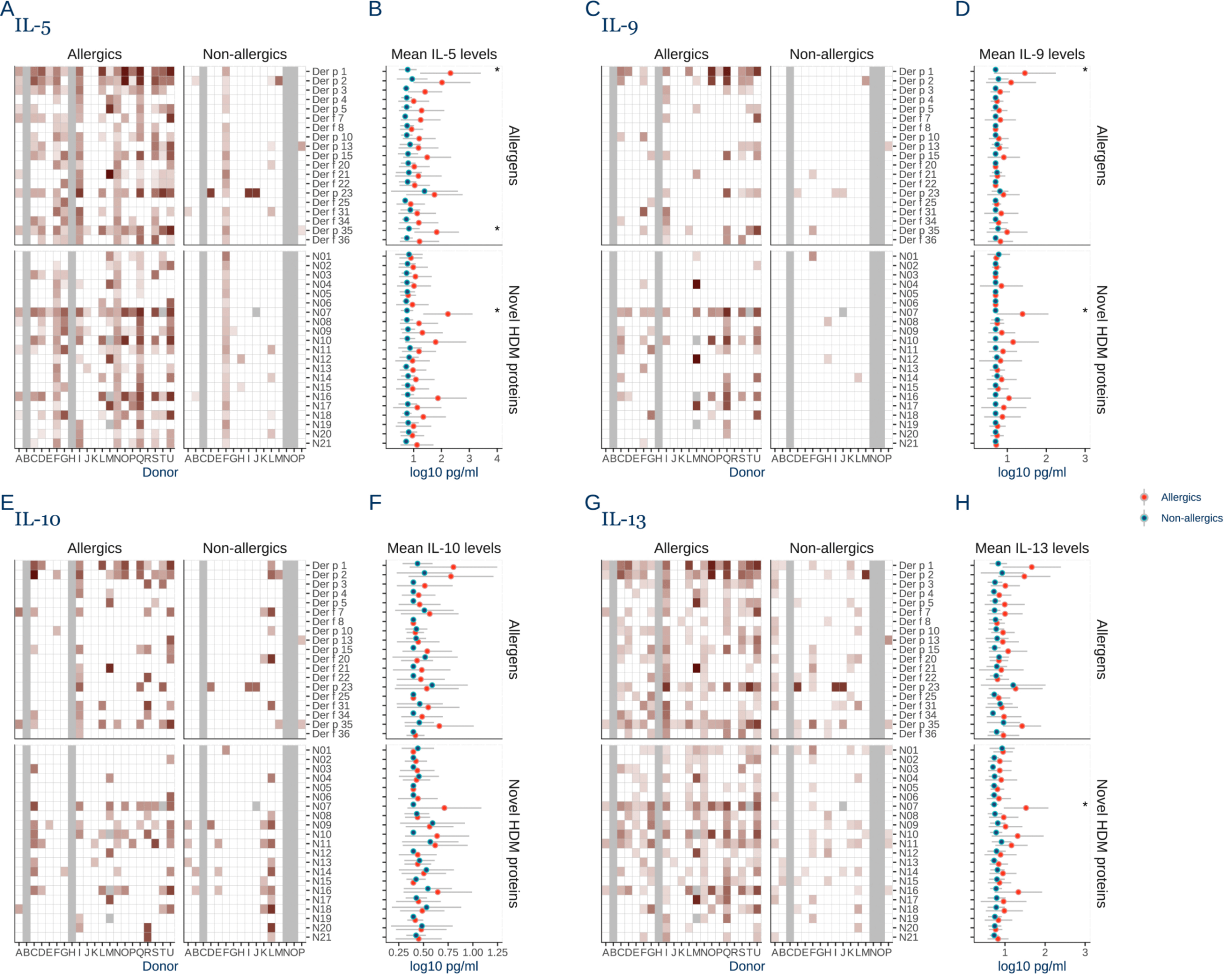
T_H_2 responses are associated with allergy and characterized by broad specificity beyond known allergens. **A)** Heatmap of IL-5 induction of 40 house dust mite (HDM) proteins in PBMCs from allergic donors (n = 19), and non-allergic donors (n = 13). IL-5 secreted from donor PBMCs stimulated for 5 days with individual HDM proteins was quantified in a Uplex assay. **B)** Mean IL-5 response to HDM proteins for allergic (red) and non-allergic donors (blue). **C)** Heatmap of IL-9 induction by the HDM proteins in allergic and non-allergic donors measured with a Uplex assay. **D)** Mean IL-9 response to HDM proteins for allergic and non-allergic donors. **E)** Heatmap of IL-10 induction by the HDM proteins in allergic and non-allergic donors measured with a Uplex assay. **F)** Mean IL-10 response to each HDM protein for allergic and non-allergic donors. **G)** Heatmap of IL-13 induction by the HDM proteins in allergic and non-allergic donors measured with a Uplex assay. H) Mean IL-13 response to each HDM protein for allergic and non-allergic donors. Error bars show the standard deviation of the log transformed values. Statistical significance was evaluated with Kruskal Wallis test, and Dunn’s post hoc test with Bonferroni correction and p-values >0.05 are indicated by *. Grey tiles denote missing data.

We observed higher cytokine induction for T_H_2 cytokines IL-5, IL-9, and IL-13, and immunosuppressive IL-10, by HDM proteins in allergic donors compared to non-allergic donors (P < 0.001). (Fig 3).

For IL-5 induction, around 80% of the allergic donors secreted IL-5 in response to Der p 1, Der p 2, Der p 35, and N07 (Fig 3A). The IL-5 induction was statistically significantly higher for allergic donors compared to non-allergic donors for Der p 1, Der p 35, and N07 (P < 0.05) (Fig 3B).

For IL-9, the most immunogenic proteins were N07 and Der p 1, which elicited responses in PBMCs from 61% and 57% of allergic donors, respectively, and the levels were statistically significantly higher compared to non-allergic donors (P < 0.001) (Fig 3C & D).

For IL-10, the most immunogenic proteins were Der p 1 and Der p 2, both eliciting positive responses in PBMCs from 53% of allergic donors, although not statistically significant different compared to non-allergic donors (Fig 3E & F).

For IL-13, we observed induction broadly across the HDM proteins in allergic donors, with the strongest positive responses by Der p 35 and N07, eliciting positive responses in 90% and 83% of donors, respectively (Fig 3G), although only N07 induction was statistically significantly higher compared to non-allergic donors (P = 0.003) (Fig 3H).

In summary, the HDM proteins broadly induced T_H_2 responses in allergic donors but only sporadic T_H_2 induction in non-allergic donors. Allergens Der p 1, Der p 2, Der p 23 and Der p35 and the novel protein N07 were the most consistent inducers of T_H_2 cytokines in allergic donors.

### HDM specific antibody levels and T-cell responses correlate only weakly to moderately

**Fig 4:**
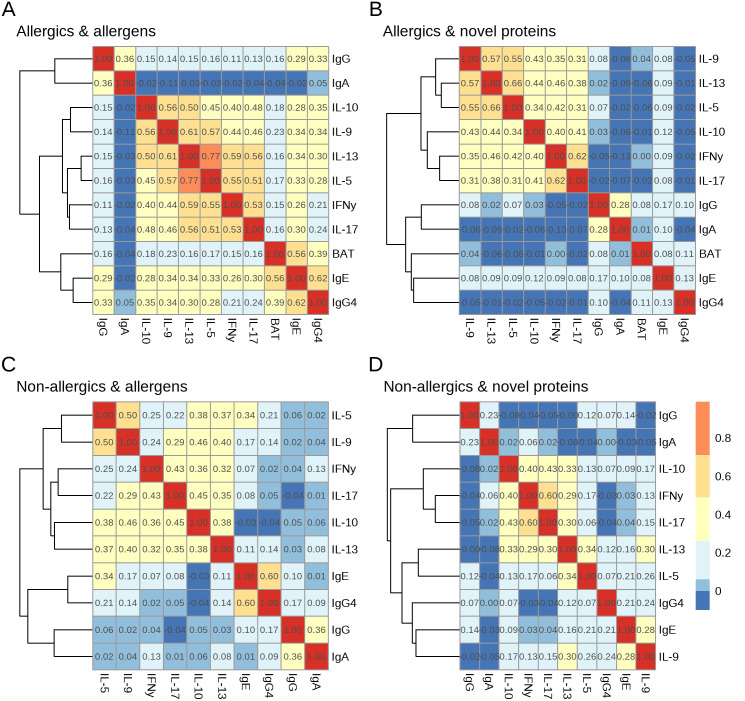
Limited correlations in strength and frequency between antibody and T-cell responses. Correlation matrix of Kendall’s tau-b correlations for **A)** Allergic donors and known house dust mite (HDM) allergens. **B)** Allergic donors and novel HDM proteins. **C)** Non-allergic donors and known allergens. **D)** non-allergic donors and novel HDM proteins. The dendrograms show the hierarchical clustering of the correlations.

Next, we correlated specific antibody repertoires, cytokine induction, and basophil activation, stratified for allergic status and protein classification. For allergic donors and allergens, there was a strong correlation between IgE and basophil activation (BAT) (tau-b = 0.56) and IgE and IgG4, (tau-b = 0.62) (Fig 4A). The cytokines were positively correlated, with the strongest correlation being IL-13 with IL-5 (tau-b = 0.77) and with IL-9 (tau-b = 0.61). IgA correlated moderately with IgG (tau-b = 0.36), while IgG also correlated moderately with IgG4 and IgE (tau-b = 0.33 and 0.29, respectively).

We observed similar patterns for novel HDM proteins in allergic donors, but here IgG4 correlated very weakly with IgE (tau-b = 0.13) (Fig 4B).

Overall correlations of non-allergic donor responses toward known allergens mirrored that of allergic donors, with IgE and IgG4 having a strong correlation (tau-b = 0.60), but induction of cytokines was less correlated (Fig 4C). For non-allergic donors and novel proteins, the highest correlation was between IFNγ and IL-17 (0.60, Fig 4D). Across all comparisons, we observed weak to moderate correlation between antibody levels and cytokine induction (maximum tau-b = 0.35 between IL-10 and IgG4).

### HDM proteins have distinct immunological profiles

To further examine the heterogeneous responses to HDM proteins, we performed a cluster analysis on the full set of immune parameters from allergic donors (Fig S2 in S1 File). Der p 2 stood out having the strongest overall IgE, IgG4, IgG and T_H_2 cytokine response. Remaining major allergens Der p 1, Der p 23, and Der p 35 clustered together inducing high levels of T_H_2 cytokines, and lower levels of specific IgG, IgE and IgG4 reactivity. N07, N09, N10, N11, and N16 induced cytokines but low antibody reactivity. Der p 10, Der f 7, N18, and Der p 15 clustered together with relatively high IgG and IgA reactivity. A large fraction of the novel HDM proteins form a cluster with largely similar overall T_H_1 and T_H_2 immunological profiles. Thus, HDM proteins appear to have distinct types of immunological profiles.

### HDM sublingual immunotherapy does not alter the overall specific antibody patterns

**Fig 5:**
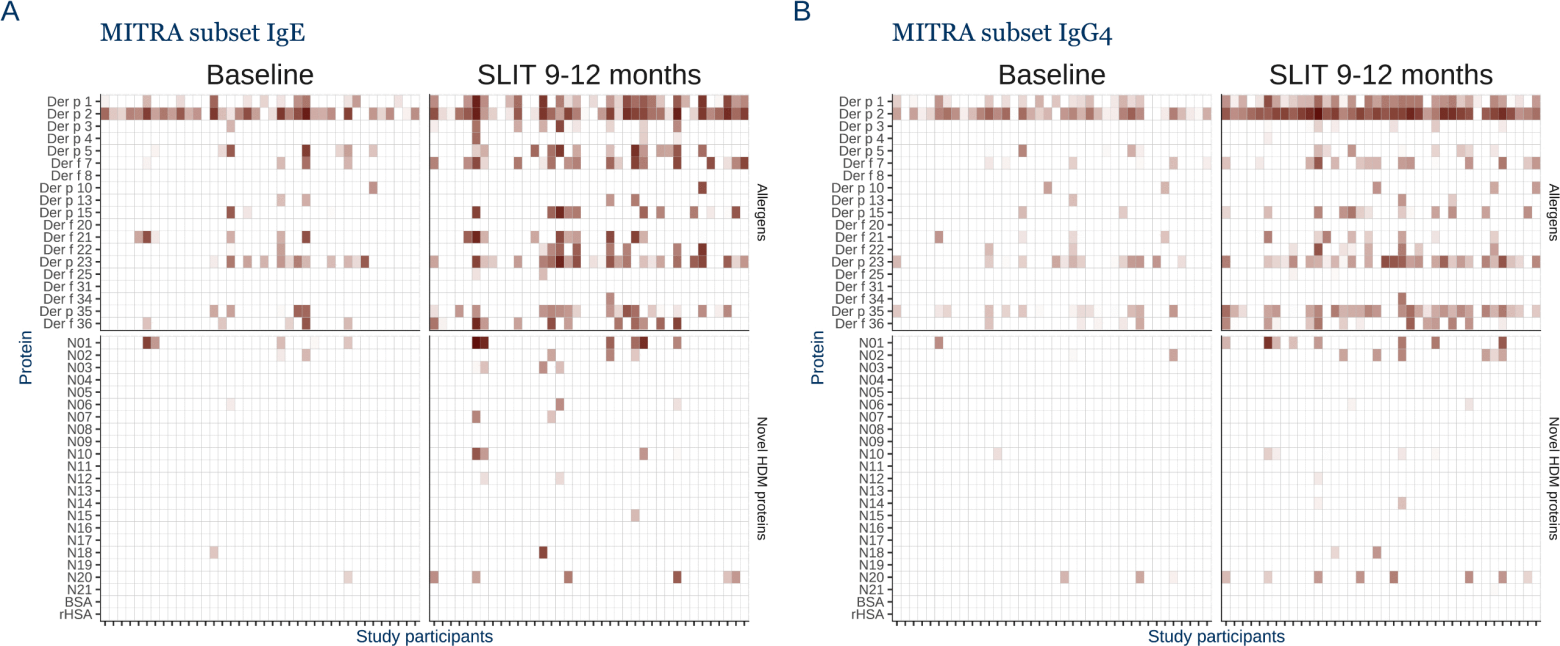
Overlap in specificity between IgE and IgG4 maintained by sublingual AIT. **A)** Heatmap of levels of specific IgE against the panel of house dust mite (HDM) proteins (n = 40) for 38 HDM allergic individuals at baseline and after 9–12 months of SQ HDM SLIT-Tablet immunotherapy (SLIT). **B)** Specific IgG4 levels against the 40 HDM proteins. Antibodies were detected with isotype specific fluorescently tagged detection antibodies, values were log 10 transformed.

Specific IgE, IgG, and IgG4 towards the HDM proteins were measured post hoc in serum samples from study participants at baseline and after 9–12 months receiving SQ HDM SLIT-tablet or placebo treatment (n = 38 and 20, respectively, Table S4 in S1 File). In accordance with the first donor cohort (Figs 1A, 2A and 2C), we observed broad IgG reactivity (Figure S3 in S1 File), and specific IgE and IgG4 to the individual HDM proteins were overlapping and predominantly targeting major allergens (Fig 5A and B, Figure S4 in S1 File).

**Fig 6:**
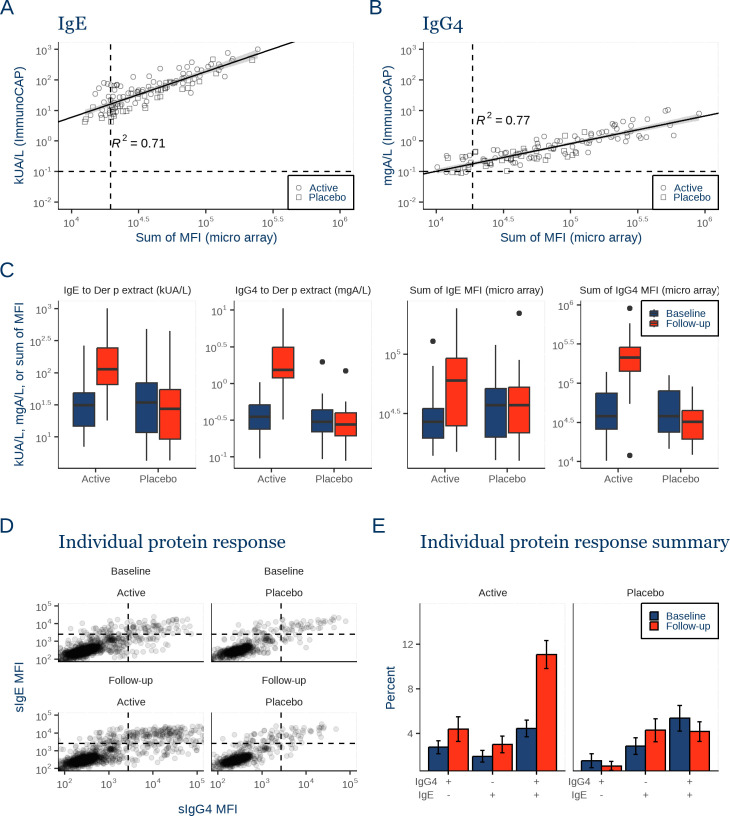
IgE and IgG4 relationship in clinical study samples. **A)** Sum of specific IgE against single HDM proteins (micro array), and Der p extract-specific IgE (ImmunoCAP), per sample. Shape indicates treatment group. Trendline indicates linear model, 95% confidence interval shown in grey. Dashed lines indicate 44 times the mean signal to BSA corresponding to the sum of background level for the micro array, and 0.1 kU/L lower limit of quantification for ImmunoCAP measurements. **B)** Sum of specific IgG4 against single HDM proteins (micro array), and Der p extract-specific IgG4 (ImmunoCAP), per sample. **C)** Specific IgE and IgG4 levels measured towards Der p extract (ImmunoCAP) and the sum of HDM protein reactivity (micro array) of active and placebo treated individuals at baseline (blue) and at follow-up (red). Boxes show median, 25th and 75th percentile levels, whiskers show highest or lowest value within 1.5 times the inter quartile range. **D)** IgE and IgG4 levels for all individual proteins for active SQ HDM SLIT and placebo treated individuals at baseline and at follow-up. Dashed lines mark the lower limit of quantification defined as the mean value of measured antibody against BSA plus 10 standard deviations. **E)** Mean percentage of measurements above the limit of quantification for each quadrant in D), error bars show the standard error of the mean (SEM).

The specific antibody levels measured with the micro array towards major HDM proteins and the sum of specific IgE towards the HDM proteins within each donor, were linearly related to the antibody levels measured to HDM extracts in a standardized quantitative assay (ImmunoCAP) above the lower limit of quantification, for both IgE and IgG4, (Fig 6A and B, Figure S6 in S1 File) and can thus be considered semi-quantitative.

Next, we examined the overall effect of SQ HDM SLIT-tablet treatment on IgE and IgG4 levels to the HDM proteins. Specific IgE levels both towards the HDM proteins and Der p extract were higher following SQ HDM SLIT-tablet treatment compared with placebo (Fig 6C). Similarly, we observed an increase in IgG4 levels both towards the HDM proteins and Der p extract (Fig 6C). As expected, no change was observed in the placebo group.

When comparing the overlap in specific IgG4 and IgE reactivity in response to SLIT treatment, we observed similar patterns of IgE and IgG4 reactivity between SQ HDM SLIT-tablet and placebo treated individuals with the majority of HDM proteins (>75%) being below detection limit (Fig 6D). For HDM proteins that are recognized by IgE or IgG4 or both, most proteins are recognized by both isotypes (Fig 6E). We observed a minor increase in the fraction of HDM proteins positive for both IgG4 and IgE reactivity following SQ HDM SLIT-tablet treatment. Together, this suggests that IgE and IgG4 specificity remain closely associated after SQ HDM SLIT-tablet treatment, with no drastic change in the overall recognition pattern of specific IgE and IgG4 across HDM proteins.

In summary, IgE and IgG4 levels to individual allergens were highly correlated, and overall pattern in HDM protein reactivity between IgE and IgG4 was maintained by SQ HDM SLIT-tablet treatment.

## Discussion

In this study, we conducted immunoproteomic profiling of a wide panel of recombinant HDM proteins in individuals with HDM allergy, non-allergic individuals, and individuals undergoing HDM sublingual tablet immunotherapy for HDM allergy. Our analysis revealed that HDM proteins were broadly immunogenic independent of allergic status, as evident by the presence of IgG, IFNγ and IL-17 responses to a broad range of HDM proteins in both allergic and non-allergic individuals. Our results were in line with earlier observations that HDM specific IgG is present in atopic and non-atopic individuals alike [26].

Overall, HDM proteins were recognized to varying degrees by different antibody types. We observed limited correlation between antibody levels and induction of T-cell responses, as measured by cytokine secretion. As expected, T_H_2 responses were strongly associated with HDM allergy. Specific IgE was mainly observed against know allergens, while T_H_2 cytokines were induced to a broad range of proteins, in line with previous findings [8]. IgE and IgG4 binding patterns were closely linked and mainly directed to major allergens, particularly Der p 2. This finding was replicated in a clinical trial cohort and further demonstrated that sublingual allergy immunotherapy maintains the specificity and overlap of IgE and IgG4 repertoires. IgG and IgA levels in non-allergic individuals were not predominantly against the major allergens Der p 1, and Der p 2. This observation echoes earlier studies, where specific IgA levels were higher towards Der p extract than towards Der p 1 and Der p 2, while specific IgE levels were skewed towards Der p 2 [14].

The presence of a broad non-T_H_2 (IgG, IFNγ and IL-17) response to HDM proteins, in allergic and non-allergic individuals, has implications for understanding immunological tolerance in allergy. Long-term efficacy of allergen immunotherapy is proposed to be associated with a shift from T_H_2 towards T_H_1 responses with induction of IgG, IgG4, and IgA blocking IgE from binding to allergens [27]. We observed lower levels of IFNγ and IL-17 induction for allergens compared to novel HDM proteins. However, the strong and allergy-independent non-T_H_2 responses suggest that non-T_H_2 responses alone are not protective against HDM allergy. Together, our data indicate that HDM allergy is primarily characterized by a robust IgE and T_H_2 response, alongside a non-pathogenic and common non-T_h_2 response. These non-T_h_2 responses might amplify the allergic inflammation in the lung tissue and contribute to the complex pathology of asthma.

It is generally accepted that frequent allergen exposure induces IgG4 [28] and that allergen immunotherapy induces allergen-specific IgG4 preventing cross-linking of receptor-bound IgE [29]. The overlapping specific IgE and IgG4 binding patterns, we report here supports the role of IgG4 as blocking IgE. A similar IgG4/IgE overlap has been observed for major grass allergens in response to subcutaneous grass immunotherapy [30] and during chronic Helminth infections [31]. The overlapping IgE and IgG4 specificity supports a common origin of the two antibody repertoires. Recent studies have identified an isotype switched memory B-cell phenotype, known as MBC2s, that retains IgE memory [32,33]. Although IgE memory does not require sequential switching through IgG1 [34], CD23 levels on IgG1 B cells correlate with serum IgE [35]. MBC2s are enriched for the IgG4 isotype and shared cellular origin of IgE and IgG4 would explain the observed overlap in specificity of IgE and IgG4 and support IgG4 belonging to the regulatory arm of T_H_2 immunity as observed in tolerant beekeepers [36].

The consistent overlap of specific IgE and IgG4 in HDM sensitized subjects (n = 58) baseline samples further supports that IgG4 is an integrated part of IgE sensitization in allergy. Increase in IgG4 levels towards Der p 1 and Der p 2 has been reported in response to SLIT [37], we similarly observed that both specific IgE and IgG4 levels increased in response to SQ HDM SLIT-tablet treatment. This increase was not associated with a change in the overall specificity pattern of IgE and IgG4 after 9–12 months of treatment. The absence of further diversification of IgE and IgG4 memory repertoires agrees with our previous analysis of IgE heavy chain variable region genes over one year of grass SLIT [38]. The rise in IgE and IgG4 to minor components in the HDM extract applied sublingually implies exposure to the relevant components and that the response to treatments is determined by the sensitization prior to treatment [13,30]. Across SLIT studies, specific IgE rises during the initial weeks, reaching peak serum levels around 8–12 weeks of treatment. Contrast, specific IgG4 increase gradually, attaining the highest levels at the conclusion of SLIT whether that is 1 year, as in this study, or 3 years as standard clinical practice [39]. The component resolved data presented here indicates that the increase in IgG4 is mainly driven by an increase in the existing IgG4 repertoire, which we hypothesize would be the same following a full 3-year treatment period.

Together this supports IgG4 as a key contributor to clinical tolerance, however, a definitive correlation between allergen specific IgG4 levels and treatment effectiveness has yet to be established. Research in this area is complicated by several confounding factors, including the high variability in patients’ immune responses, the complexity of patient-reported outcomes in clinical trials, and the presence of multiple contributing mechanisms. One such mechanism is desensitization, which occurs rapidly upon allergen exposure, suggesting it is unlikely to be directly linked to IgG4. Tolerance and sustained effects has been reported after 1 year of treatment [17] although it may not yet be fully developed. Additionally, distinct phases of longitudinal changes in B and T-cells have been reported during SLIT treatment [40], but we did not have PBMCs from the SQ HDM SLIT-tablet treated individuals to evaluate immunomodulatory effect of SLIT in this study.

Component-resolved measurement of allergen specific IgG4 and IgE can help disentangle the antibody responses. Through unsupervised clustering, we identified Der p 2 as a distinct immunodominant cluster, showing the highest IgE and IgG4 levels and pronounced T_H_2 and non-T_H_2 immunogenicity. This underscores Der p 2’s structural or functional allergenic features. Its capacity to bind TLR4 and LPS may influence T_H_2 immunity through innate pathways [41]. For Non-T_H_2 responses to Der p 2, only IgG was associated with allergy. This was not solely due to increased IgG4, as Der p 2 specific IgG1 was observed (Figure S5 in S1 File), consistent with the observation that IgG1 Memory B-cells precede IgE plasma cells [42]. The HDM proteins investigated in this study, are likely released to the mucosa simultaneously, pointing to other factors, such as intrinsic protein immunomodulatory features or cross-reactivity to homologous molecules from other allergenic species, as main drivers of allergenicity.

In our Danish donor cohorts, it was not possible to evaluate the specific levels of HDM exposure. However both allergic and non-allergic donors resided in the greater Copenhagen area. The allergic and non-allergic group were closely matched in age, but the gender composition differed between the two groups. We detected specific IgE towards HDM proteins and HDM extract in one non-allergic donor (non-allergic donor F), who exhibited no allergic symptoms and could therefore be considered sub-clinically sensitized. While IgE against allergens is a hallmark of allergic sensitization, not all IgE positive individuals develop symptoms [43]. The inclusion of potentially sensitized individuals in the non-allergic control group did however not impact the overall conclusions presented here.

The response to allergens in allergic individuals has been studied extensively. This study extends well beyond known allergens and includes a non-allergic reference group, and unbiased analysis of correlations between responses. In conclusion, our findings show several novel features of HDM allergy: HDM proteins are commonly and broadly immunogenic independent of allergy status, but T_H_2 responses are restricted to allergic subjects. IgE and IgG4 repertoires appear to co-evolve and overlap in specificity. This has implications for understanding the pathology of HDM allergy and the mode of action of allergen immunotherapy. Following the immunological patterns longitudinally, starting in early childhood, might help us better understand HDM allergic disease, and reveal optimal time points for therapeutic interventions.

### Key messages

- Both allergic and non-allergic individuals display immunogenic responses to a broad range of HDM proteins.
- TH2 responses, especially against Der p 2, and overlapping IgG4 and IgE repertoires differentiate allergic individuals.
- IgE/IgG4 overlap is maintained during sublingual immunotherapy.

## Supporting information

S1 File Supporting methods, tables, and figures. (DOCX)

S2 File Minimal data set. (XLSX)
