# Supplementary material for: Broad immunogenicity of house dust mite proteins contrasts restricted specific IgE and IgG4 associated with allergy
Source: PLoS One. 2026 Feb 9;21(2):e0338593. doi: 10.1371/journal.pone.0338593 (PMC12885264; doi:10.1371/journal.pone.0338593)
Supplement: S1 File — (DOCX) [file pone.0338593.s001.docx]

**Supplemental information:**

**Table of contents**

Materials and Methods

- Gyrolab IgG1 and IgG4 measurements

Supplemental figures

Table S1. Protein expression

Table S2. Biochemical function and homology of novel HDM proteins

Table S3. Danish cohort

Table S4. MITRA cohort

Supplemental figure S1: SDS-PAGE of purified recombinant HDM proteins.

Supplemental figure S2: Allergen heatmap plot

Supplemental Figure S5: IgG1 & IgG4 Der p 2 heatmap

Supplemental Figure S3: MITRA trial subset- specific IgG heatmap, before and after treatment

Supplemental Figure S4: Specific IgE and IgG4 for placebo treated MITRA trial participants.

Supp table 2: Biochemical function and homology of novel HDM proteins

**Methods**:

**Gyrolab IgG1 and IgG4 measurements**

The serum levels of IgG4 and IgG1 specific for Der p 2 were determined with an anti-human IgG1 or IgG4 immunoassay using Gyrolab microfluidic CD technology (Gyros, Sweden). Briefly, biotinylated Der p 1 or Der p 2 was used to capture IgG1 or IgG4 in serum. After a wash step, an AF 647-labeled anti-human IgG1 (Southern Biotechnology (ahIgG1) or IgG4 (Thermo Fischer Scientific) specific antibody was used to detect bound sIgG1 or sIgG4 in the unknown samples. The Fluorescence intensities were then extrapolated on a calibrator curve generated with a recombinant IgG1 or IgG4 specific against either Der p 1 or Der p 2.

**Supplemental figures & tables:**

**Table S1.** Protein expression

| **Expression system** | **Protein** | **Endotoxin (EU/µg)** |
| --- | --- | --- |
| **HEK293** | Der p 2.0101 | <0.0021 |
|  | Der p 3.0101 | <0.0008 |
|  | Der p 4.0101 | <0.0005 |
|  | Der p 10.0101 | <0.0013 |
|  | Der p 13.0101 | <0.0012 |
|  | Der p 15.0102 | <0.0014 |
|  | Der f 21.0101 | <0.0009 |
|  | Der f 22.0101 | <0.0014 |
|  | Der f 31.0101 | <0.0018 |
|  | Der f 34.0101 | <0.0025 |
|  | Der p 35* | <0.0005 |
|  | Der f 36.0101 | <0.0056 |
|  | N01 | <0.0001 |
|  | N02 | <0.0009 |
|  | N03 | <0.0003 |
|  | N05 | <0.0011 |
|  | N06 | <0.0001 |
|  | N07 | <0.0006 |
|  | N11 | <0.0042 |
|  | N12 | <0.0002 |
|  | N20  N21 | <0.0004  <0.0001 |
| **Yeast/P. pastoris** | Der p 1.0105 | <0.0003 |
| **E.coli** | Der p 5.0101 | <0.0069 |
|  | Der f 7.0101 | <0.0080 |
|  | Der f 8.0101 | <0.0114 |
|  | Der f 20.0201 | <0.0036 |
|  | Der p 23.0101 | <0.0036 |
|  | Der f 25.0201 | <0.0028 |
|  | N08 | <0.0040 |
|  | N09 | <0.0023 |
|  | N10 | <0.0037 |
|  | N13 | <0.0004 |
|  | N14 | <0.0012 |
|  | N15 | <0.0012 |
|  | N16 | <0.0062 |
|  | N17 | <0.0034 |
|  | N18 | <0.0007 |
|  | N19 | <0.0071 |
| **Sf9** | N04 | <0.0020 |

***** Currently, Der f 35.0101 is the only group 35 HDM allergen recognized by IUIS, and hence, "Der p 35" was initially a novel protein when we identified this in our previous study (Osserof et al., 2016) but seems to be a Der p homolog of Der f 35. Thus, it shows 99.2 % identity to "Der f 35-like allergen" (ATI08948) and 84.9 % identity to Der f 35.0101 (93.7 % homology when differences in homologous amino acids are excluded). It seems to be a distant proteoform/isoform of Der p 2 as previously described by Fujimura et al.^7^

**Table S2**. Biochemical function and homology of novel HDM proteins

| HDM protein | Derived  from | Closest homolog: Biochemical function and [species]* | Accession ID | Identity to Der p or f^ |
| --- | --- | --- | --- | --- |
| N01 | Der f | 100 %, uncharacterized protein LOC124493199 [Der f] | XP_046912236 | Der p: 87 % |
| N02 | Der f | 99.2 %, fatty acid-binding protein-like [Der f]  80.3 %, Group 13 mite allergen-like protein (Lipocalin) [Eur m] | XP_046909322  OTF83183 | Der p: 83 % |
| N03 | Der p | 99.1 %, carboxypeptidase Q-like [Der p] | XP_027206047 | Der f: 90 % |
| N04 | Der f | 100 %, fructose-bisphosphate aldolase-like isoform X2 [Der f] | XP_046919606 | Der p: 94 % |
| N05 | Der f | 100 %, nucleoside diphosphate kinase B-like [Der f] | XP_046914899 | Der p: 99% |
| N06 | Der p | 99.2%, lysosomal alpha-mannosidase-like [Der p] | XP_027202659 | Der f: 81 % |
| N07 | Der p | 98.8%, lysosomal alpha-glucosidase-like, partial [Der p] | XP_027203752 | Der p: 74 % |
| N08 | Der f | 100%, pantetheinase-like [Der f] | XP_046915387 | Der p: 84 % |
| N09 | Der f | 98.6%, chitinase-3-like protein 1 [Der f] | XP_046911662 | Der p: 86 % |
| N10 | Der f | 99.3%, dipeptidyl peptidase 1-like [Der f] | XP_046909289 | Der p: 72 % |
| N11 | Der p | 99.8%, acid phosphatase type 7-like [Der p] | XP_027200750 | Der f: 82 % |
| N12 | Der p | 97.8%, peptidase 1-like [Der p] | XP_027203088 | Der f: 58 % |
| N13 | Der f | 100%, glyceraldehyde-3-phosphate dehydrogenase 2-like [Der f] | XP_046912585 | Der p: 97 % |
| N14 | Der f | 100%, uncharacterized oxidoreductase MexAM1_META1p0182-like [Der f] | XP_046920616 | Der p: 78 % |
| N15 | Der f | 100% glutathione transferase delta-like [Der f] | KAH7641750 | Der p: 83 % |
| N16 | Der p | 100%, beta-1,3 glucanase Glu1 [Der p] | QER90682 | Der f: 89 % |
| N17 | Der f | 100%, glutamate receptor ionotropic, kainate 2-like [Der f] | XP_046908642 | Der p: 82 % |
| N18 | Der f | 100%, filamin-A-like [Der f] | XP_046908591 | Der p: 97 % |
| N19 | Der f | 99.6% uncharacterized protein LOC124499317 [Der f] | XP_046919164 | Der p: 51 % |
| N20 | Der p | 99.4%, uncharacterized protein LOC113796509 [Der p]  74.4 %, cathepsin B-like [Der f] | XP_027202599  XP_046918597 | Der f: 74 % |
| N21 | Der p | 100%, calreticulin-like [Der p] | XP_027196726 | Der f: 94 % |

*BLAST® NCBI

^Sequence identity to homolog HDM species (Der p or f) either by searching in-house transcriptomes or NCBI

**Table S3**. Danish donor cohort: Allergic and non-allergic donors

|  | Allergic (N=21) | Non-allergic (N=16) | Total (N=37) | p value |
| --- | --- | --- | --- | --- |
| **Age** |  |  |  | 0.889 |
| Mean (SD) | 48.0 (14.4) | 47.3 (17.4) | 47.7 (15.5) |  |
| Range | 23.0 - 70.0 | 21.0 - 67.0 | 21.0 - 70.0 |  |
| **Gender** |  |  |  | 0.004 |
| Female | 14 (66.7%) | 3 (18.8%) | 17 (45.9%) |  |
| Male | 7 (33.3%) | 13 (81.2%) | 20 (54.1%) |  |

**Table S4**. MITRA subset cohort:

|  | Active (N=38) | Placebo (N=20) | Total (N=58) | p value |
| --- | --- | --- | --- | --- |
| Age |  |  |  | 0.212 |
| Mean (SD) | 31.3 (8.8) | 34.6 (11.3) | 32.4 (9.8) |  |
| Range | 19.0 - 52.0 | 20.0 - 53.0 | 19.0 - 53.0 |  |
| Gender |  |  |  | 0.349 |
| F | 16 (42.1%) | 11 (55.0%) | 27 (46.6%) |  |
| M | 22 (57.9%) | 9 (45.0%) | 31 (53.4%) |  |
| Total IgE |  |  |  | 0.270 |
| Mean (SD) | 34632.8 (24046.2) | 42753.8 (30390.3) | 37433.1 (26426.3) |  |
| Range | 13873.3 - 128881.5 | 12773.9 - 119328.8 | 12773.9 - 128881.5 |  |
| Der p IgE |  |  |  | 0.386 |
| Mean (SD) | 46.1 (51.6) | 64.1 (105.6) | 52.3 (74.3) |  |
| Range | 7.0 - 264.2 | 4.2 - 478.3 | 4.2 - 478.3 |  |
| Der p IgG4 |  |  |  | 0.922 |
| Mean (SD) | 0.4 (0.3) | 0.4 (0.4) | 0.4 (0.3) |  |
| Range | 0.1 - 1.0 | 0.1 - 2.0 | 0.1 - 2.0 |  |


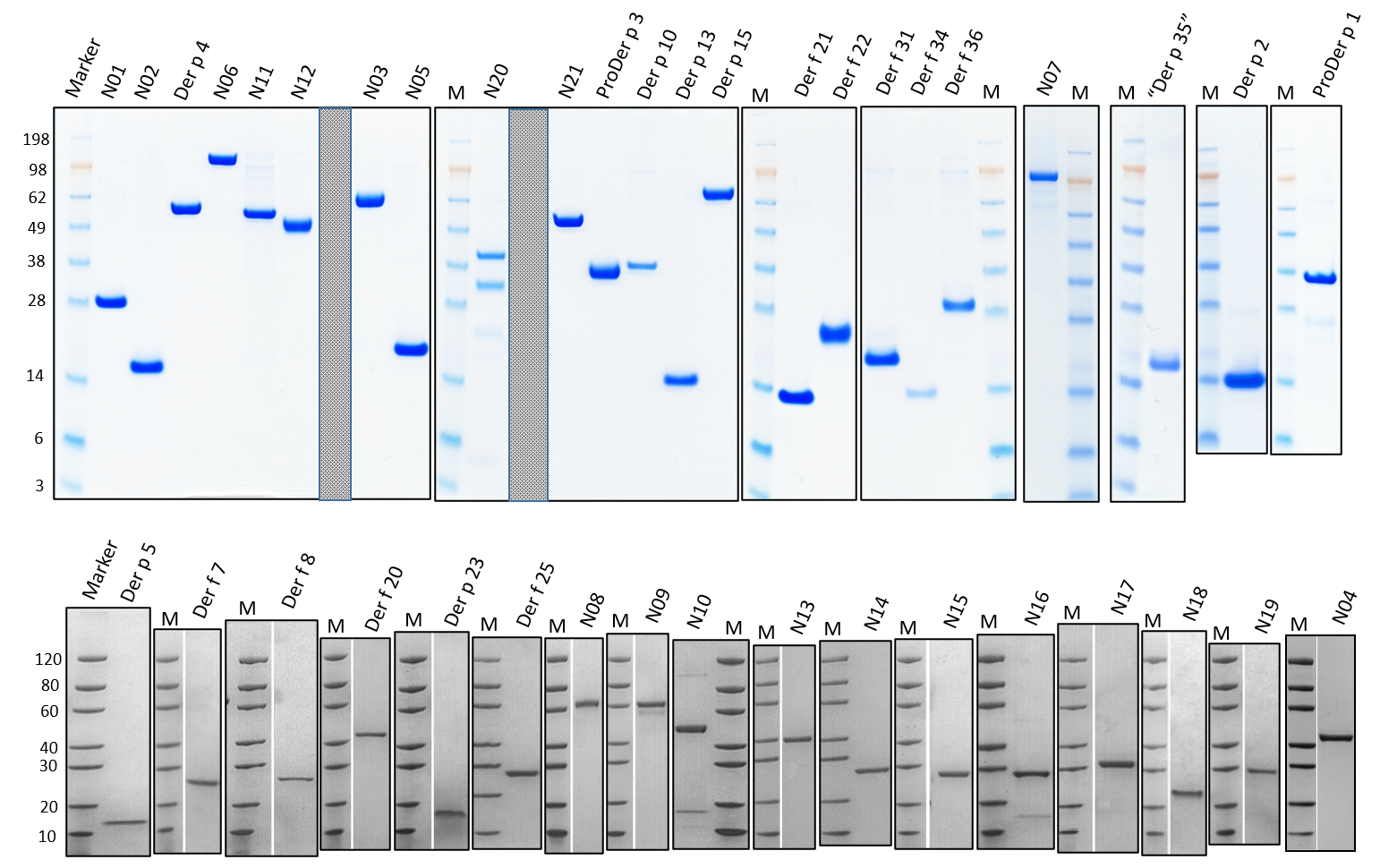


**Figure S1.** SDS-PAGE of purified recombinant HDM proteins.

Upper panel: Proteins produced in-house (HEK293 + P. pastoris)

Lower panel: Proteins obtained from Genscript (E.coli + Sf9)

*
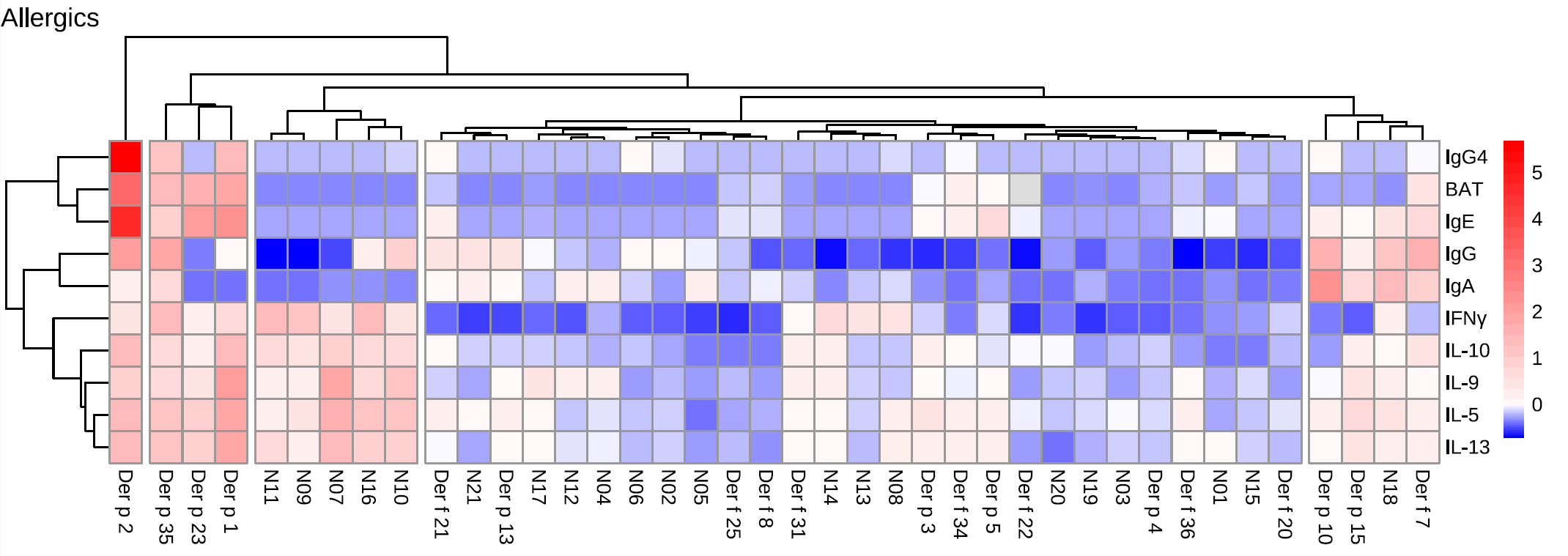
* **Figure S2*.*** **Individual proteins had distinct immunological profiles including the four major allergens.**Hierarchical clustered heat map of mean levels of reactivity to the individual HDM proteins in allergic donors as Z-score for each assay. 5 clusters of HDM proteins were defined based on the pattern of response across assays.


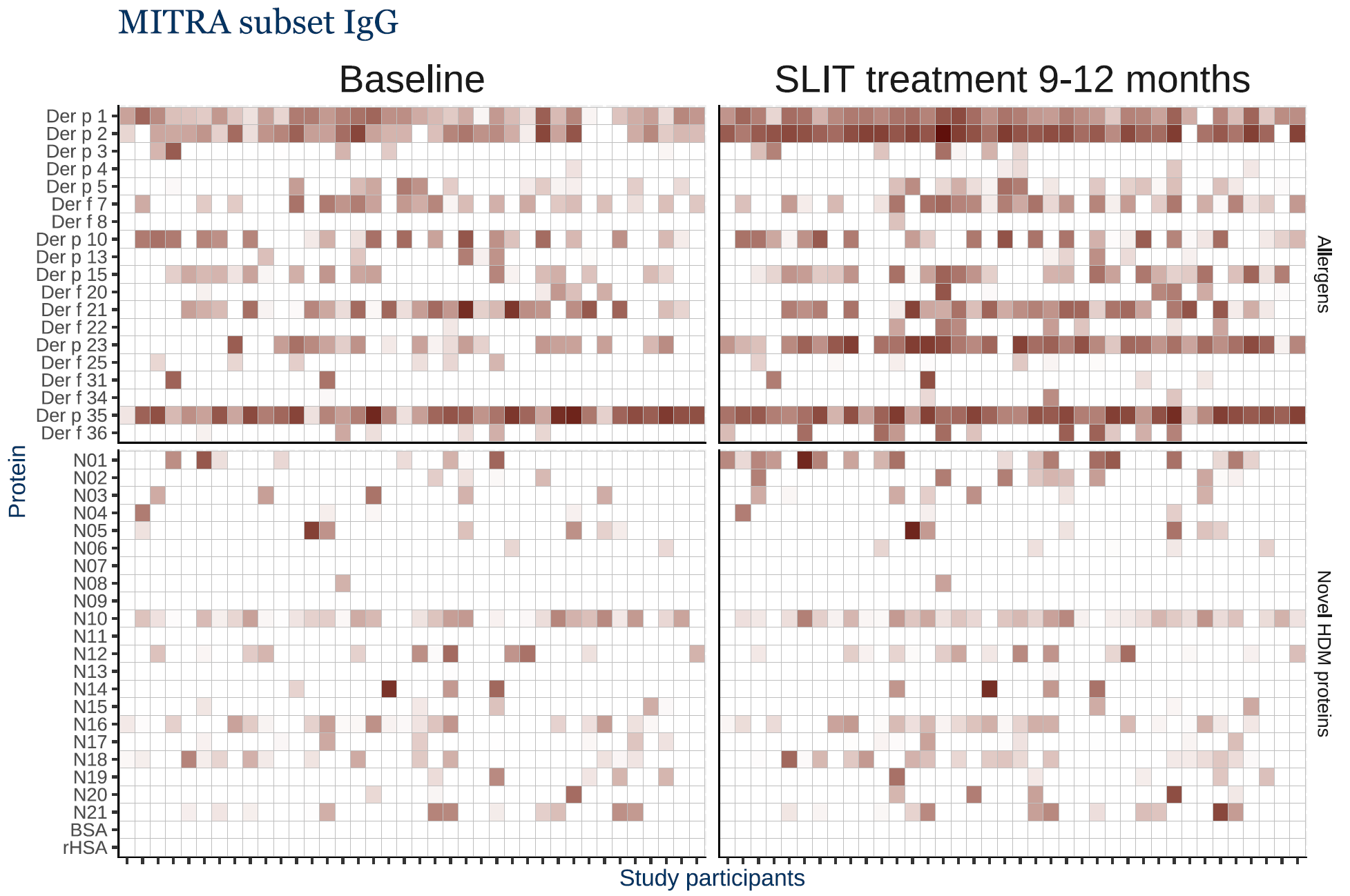


**Figure S3.** Specific IgG levels against the panel of 40 HDM proteins before and after 9-12 months of SQ HDM SLIT-Tablet immunotherapy (SLIT). Lower limit of quantification is defined as mean BSA values + 10 standard deviations.


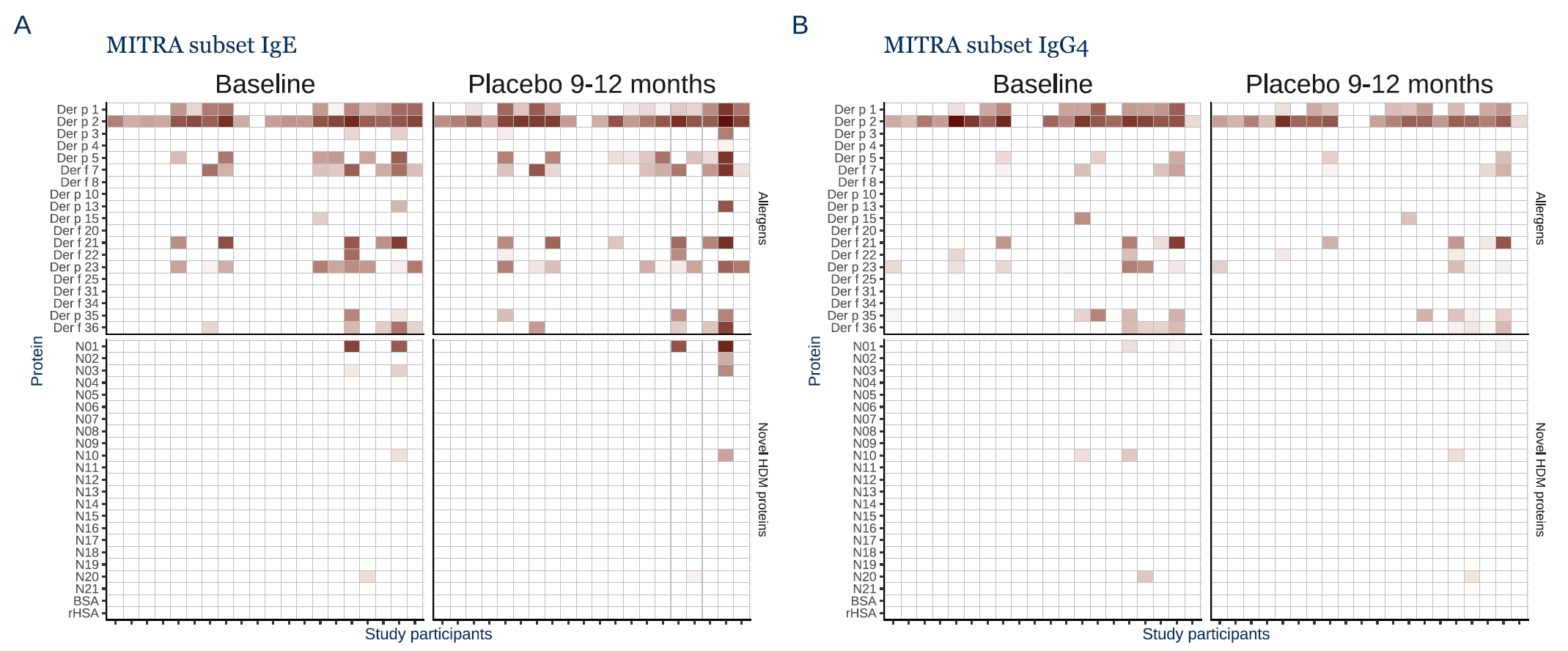


**Figure S4. A)** Heatmap of levels of specific IgE against the panel of house dust mite (HDM) proteins (n = 40) for 20 HDM allergic individuals at baseline and after 9-12 months of placebo treatment. **B)** Specific IgG4 levels against the 40 HDM proteins. Antibodies were detected with isotype specific fluorescently tagged detection antibodies, values were log10 normalized.


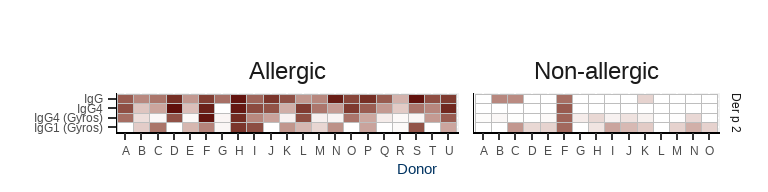


**Figure S5.** Comparison of IgG, IgG4, and IgG1 levels against Der p 2 in allergic and non-allergic donors measured with the micro-array assay (IgG and IgG4) and with GyroLab (IgG4 –“IgG4 (Gyros)” and IgG1 – “IgG1 (Gyros)”)


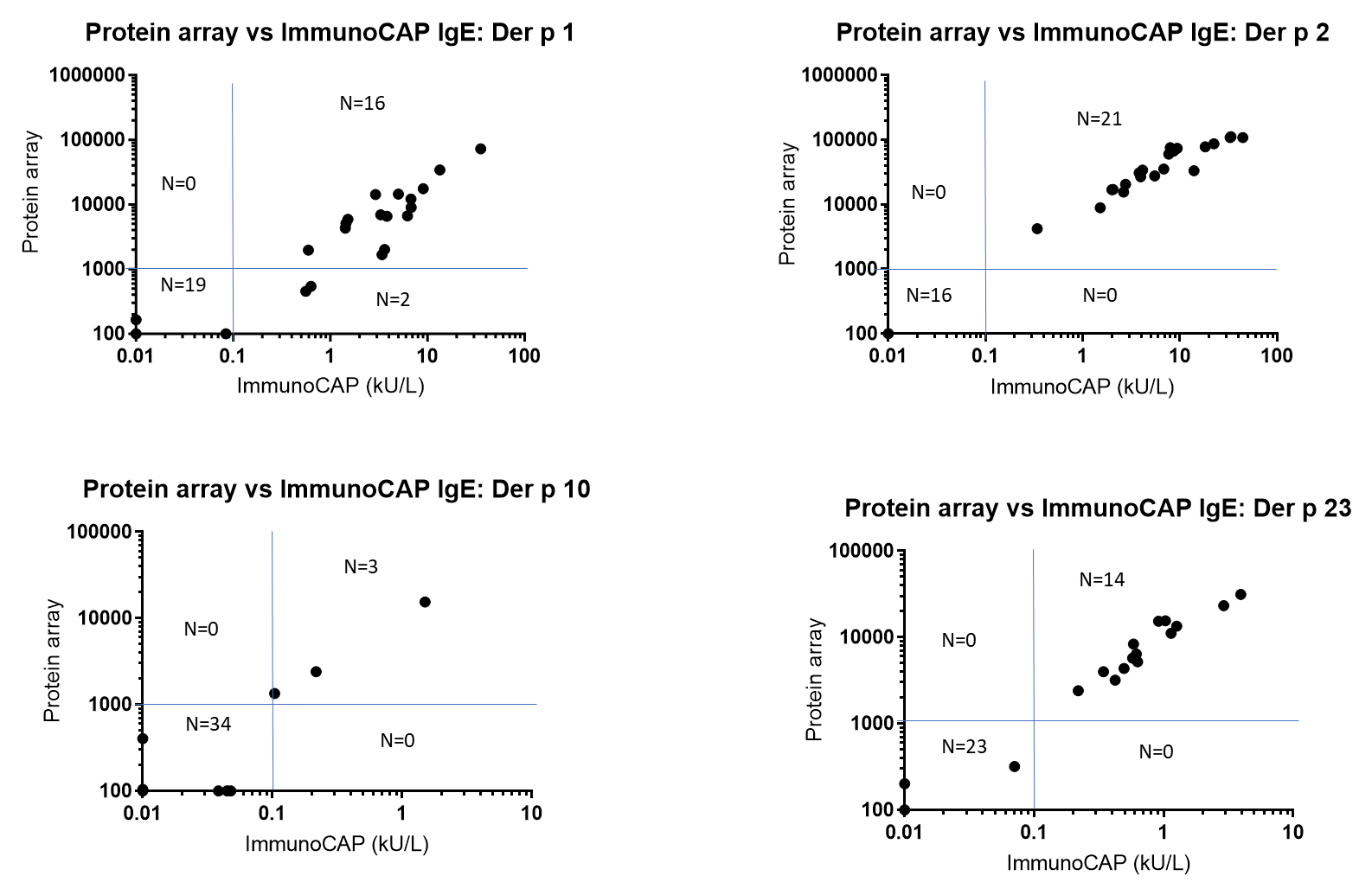


Figure S6 Correlation plots - Micro array vs ImmunoCAP IgE.
